# The Tissue-Selective Estrogen Complex (Bazedoxifene/Conjugated Estrogens) for the Treatment of Menopause

**DOI:** 10.1155/2017/5064725

**Published:** 2017-12-05

**Authors:** Stefano Lello, Anna Capozzi, Giovanni Scambia

**Affiliations:** Department of Woman and Child Health, Policlinico Gemelli Foundation, Largo Agostino Gemelli, Roma, Italy

## Abstract

The tissue-selective estrogen complex (TSEC) pairs conjugated estrogens (CE) with a selective estrogen receptor modulator (SERM), bazedoxifene acetate (BZA). A 2-year treatment with the TSEC improved vasomotor symptoms, quality of life, and vaginal atrophy in healthy postmenopausal women. In addition, the TSEC prevented vertebral and hip bone loss without increasing mammographic density, breast tenderness, the risk of myocardial infarction, stroke, or venous thromboembolism. Finally, the BZA 20 mg/CE 0.45 mg dose did not increase the risk of endometrial hyperplasia. Based on these findings, the TSEC can be considered as a first-line treatment for symptomatic postmenopausal women.

## 1. Introduction

Traditional treatments for the management of menopause and postmenopause include hormone replacement therapy with estrogen and progestin (HT) or, for hysterectomized women, estrogen therapy (ET, with estrogen only). Other options are selective estrogen receptor modulators (SERMs) or condition-specific treatments, such as bisphosphonates, denosumab, or teriparatide for osteoporosis. It is widely accepted, as shown by the Women's Health Initiative (WHI), that using conjugated estrogens together with progestin (medroxyprogesterone acetate) in women with no prior hysterectomy to protect them from endometrial hyperplasia [1] is associated with an increased—although moderate (classified as “rare” < 1/1000 per year)—risk of developing breast cancer [2]. However, treatment with conjugated estrogens reduces the breast cancer risk in women with prior hysterectomy (7 fewer cases in 100,000 per year) [3].

All the above-mentioned therapies can act as agonists or antagonists of estrogen receptors based on their chemical composition. From a clinical point of view, this translates in tissue-specific responses. For example, SERMs have estrogen agonist effects on bone tissue and antiestrogen effects on the breast and endometrium [4]. In particular, the only SERM indicated for vulvar-vaginal atrophy (VVA) is ospemifene [5].

Hence, based on the class of the molecule under study, the effects exerted on target tissues can be different, depending on the pharmacological action of single molecules or combination therapies (Table 1). The tissue-selective estrogen complex (TSEC) conjugates a SERM (20 mg bazedoxifene, BZA) with an estrogen compound (conjugated estrogens (CE), 0.45 mg/day). The TSEC has been shown to have positive effects on hot flushes (and, in general, on menopausal symptoms), vagina, bone tissue, libido, and energy levels and neutral effects on the breast and uterus [10, 11].

An ideal TSEC should, in fact, display the positive effects demonstrated by single compounds without having, or at least, minimizing any possible undesirable side effects [12].

Estrogens act on many tissues since both forms of estrogen receptors (ER-*α* and ER-*β*) are differently expressed throughout the body.

Indeed, classical target tissues for estrogen action include the brain (in which ER-*α* and ER-*β* are expressed), [13, 14], uterus (mainly ER-*α* expression) [15], bone (ER-*α* and ER-*β* expression) [16, 17], breast (ER-*α* and ER-*β* expression) [18], ovaries (ER-*α* and ER-*β* expression) [14], and liver (ER-*α* expression) [19]. The chemical structure of CE, the estrogen component of the TSEC, is characterized by B-ring-saturated molecules (classical estrogens) that act predominantly on ER-*α* and B-ring-unsaturated molecules (delta7-estrogen, delta6-8-estrogen, and delta8-estrogen) that act mainly on ER-*β*, originating an intrinsically balanced receptor modulation; of note, all CE are antioxidants [20].

As stated above, SERMs can bind to estrogen receptors [8, 21], with mixed agonist/antagonist activity depending on the genes and tissues being targeted. SERMs can mimic estrogen activity in some cases (agonist effect) and can inhibit estrogen action in other (antagonist effect) [22]. For this reason, SERMs have tissue-specific activity and can be ranked on an agonist/antagonist activity continuum [11].

The aim of this review was to summarize recent preclinical and clinical data on the action of the TSEC. We performed literature searches with PubMed for articles published in English after 2002 using the following keywords: TSEC, bazedoxifene, conjugated estrogens, HT, menopausal symptoms, quality of life, sexuality, bone, cardiovascular system, and oncologic risk.

## 2. TSEC: Preclinical Data on Compounds Used Alone or in Association (Tables 2 and 3)

### 2.1. Continuum of Antagonist/Agonist Activity: Selective Modulation of the Proliferation of Breast Cells (Cancer and Normal) in *In Vitro* Studies Using Animal Models (Table 2)

BZA shows the best antagonist activity on the proliferation of breast cancer cells (MCF-7) according to the activity ranking of SERMs [23–25, 31].

As monotherapy, SERMs alone (either BZA, raloxifene, or tamoxifen) do not induce an antagonist or agonist effect on the proliferation of MCF-7 cells [25, 31]. However, in a study with MCF-7 cancer cells treated with CE in addition to single SERMs, all the molecules tested (BZA, raloxifene, and lasofoxifene) exerted an antagonist effect on the estrogen-induced MCF-7 cell proliferation. The activity ranking established for SERMs in MCF-7 cells was BZA > raloxifene > lasofoxifene [25].

In addition, BZA was more effective than RLX and LAS in reducing ductal growth following estrogen stimulation in ovariectomized mice [26].

### 2.2. Selectivity and Endometrial Effects of SERMs: *In Vivo* Studies Using Animal Models

The action of different SERMs on the endometrial tissue can be assessed by their ability to increase uterine wet weight in various animal models. These data can be used to establish an agonist/antagonist activity ranking of SERMs in the endometrium.

BZA, either as monotherapy or in association with estrogens, induces the smallest effect on uterine wet weight in immature rats and ovariectomized mice or rats in comparison with controls treated with vehicle or with raloxifene and lasofoxifene [8, 22, 32].

Raloxifene is a more potent antagonist than lasofoxifene [8, 26, 32]. Tamoxifen was shown to significantly increase uterine wet weight in ovariectomized rats, in comparison to control, to a relatively greater extent than raloxifene but not as much as 17 *β*-estradiol. Furthermore, the uterine wet weight of ovariectomized immature mice was measured six hours after treatment with estrogens or SERM. In this study, BZA acted as an antagonist on the uterus since it did not increase uterine wet weight in comparison with control (vehicle). On the contrary, both raloxifene and lasofoxifene induced an increase in uterine wet weight compared to control. The antagonist effect on the uterus was higher with BZA, lower with raloxifene, and even lower with lasofoxifene [26].

BZA facilitates the degradation of ER-*α* on the uterus and breast [24, 30], exerting an antiestrogenic effect through the negative modulation of the receptor *α*.

Overall, preclinical data show how the TSEC compounds bind to ER and how different SERMs have different effects on target tissues. The TSEC (BZA/CE) does not stimulate the endometrium (contrary to what happens with estrogens used alone or in association with some SERMs) nor the breast (as reported with estrogen and progestin therapy) [24, 26–28, 30].

### 2.3. Bone Tissue Selectivity of SERMs: *In Vivo* Studies Using Animal Models

According to the activity ranking of SERMs in bone tissue, BZA, raloxifene, lasofoxifene, and tamoxifen all act as agonists, at different doses, and preserve bone mineral density (BMD) [8, 22, 29, 33, 34].

BZA preserves bone mass in an osteopenic rat model after six weeks of treatment with 0.3 mg/kg, a dose ten times inferior to the effective dose of raloxifene (3 mg/kg/day), but higher than the necessary dose of estradiol (2 *μ*g) [8]. Kharode et al. showed that BZA (3.0 mg/kg/day) significantly increases bone mass in ovariectomized rats in comparison with vehicle control and that the skeletal response is analogous to that obtained with CE (2.5 mg/kg/day) [29]. Lasofoxifene and raloxifene showed agonist activity in bone tissue similar to that seen with tamoxifen [33].

## 3. TSEC: Clinical Trials

Clinical trials designed to assess the activity of the TSEC have been performed on more than 7500 women around the world [1–4, 6], in a series of studies named SMART (Selective estrogens, Menopause, And Response to Therapy) [35–40].

### 3.1. Effects on Vasomotor Symptoms

The safety and efficacy of BZA 20 mg/CE 0.45 mg, the only authorized and clinically available dose in Italy, for the treatment of vasomotor symptoms in menopause were evaluated in the randomized, multicenter, double-blind, placebo-controlled SMART-1 clinical trial [35]. The SMART-1 enrolled 3397 healthy postmenopausal women, aged from 40 to 75 years, with an intact uterus. The results from SMART-1 show that both BZA 20 mg/CE 0.625 and BZA 20 mg/CE 0.45 mg significantly decrease the frequency and severity of hot flushes (HFs) in comparison with placebo. At week 12, the number of HFs was reduced by 51.7% to 85.7% with all doses and by 17.1% with placebo (*p* < 0.01). The effect on HFs was also observed during the randomized, double-blind, placebo-controlled SMART-2 trial [36] that lasted for 12 weeks. The SMART-2 trial was conducted in 318 healthy postmenopausal women with an intact uterus; 127 women were treated with BZA 20 mg/CE 0.45 mg and 63 with placebo. Inclusion criteria defined menopause as at least 12 months of amenorrhea or 6 months of amenorrhea with levels of serum follicle stimulating hormone greater than 40 mIU/mL. The mean age of the women enrolled in the study was 53 years (range: 42–64 years); mean years since menopause onset was 4.5 years; all women had natural menopause. Women had at least seven moderate or severe daily HFs or at least 50 weekly HFs at screening. Primary efficacy endpoints were changes from baseline of the mean number and score of daily HFs at weeks 4 and 12. Patients recorded HFs-related data on a daily diary card during screening and the entire period of the trial [37]. A statistically significant decrease in the frequency and severity of HFs was seen starting from week 4 in women treated with BZA 20 mg/CE 0.45 mg compared to placebo (*p* < 0.001), which demonstrates the rapid action of TSEC.

### 3.2. Effects on Bone Tissue

The efficacy of the TSEC for the prevention of osteoporosis was assessed in two clinical trials (SMART-1 and SMART-5). The SMART-1 trial [41] enrolled 2315 healthy postmenopausal women (mean age: 56 years; range: 40–75 years) assigned to two substudies. Substudy I (*n* = 1454) included women in menopause for more than five years who were osteopenic and had at least one additional osteoporosis risk factor. Substudy II (*n* = 861) enrolled women with less than five menopausal years with at least one additional osteoporosis risk factor. The endpoint was BMD changes as assessed by dual-energy X-ray absorptiometry (DXA) at the lumbar spine and total hip. A significant increase from baseline of mean BMD at the lumbar spine and hip was reported after 24 months of treatment with BZA 20 mg/CE 0.45 mg (as well as with another dose investigated in the study: BZA 20 mg/CE 0.625 mg) in women in menopause for more than five years. Also, there was a statistically significant difference (*p* < 0.01) compared with placebo at each time point (6, 12, and 24 months). A similar result was reported at the lumbar and total hip of women 1–5 years into postmenopause. In addition, there were differences in BMD between treatment groups (BZA 20 mg/CE 0.45 mg or BZA 20 mg/CE 0.625 mg) and placebo at each time point (*p* < 0.01 at 6, 12, and 24 months). Changes in bone metabolism markers assessed in the SMART-1 trial (osteocalcin as bone formation marker and C-telopeptide as serum resorption marker) were significantly increased following treatment with all BZA/CE doses compared with placebo (*p* < 0.001). The study investigators concluded that BZA/CE decreases bone turnover and bone loss in postmenopausal women with increased osteoporosis risk.

These data were confirmed by the one-year, double-blind, randomized, placebo-controlled SMART-5 clinical trial [42], which enrolled 1886 women (mean age: 53 years; range: 40 to 60 years) postmenopausal for 1–5 years and with at least one additional osteoporosis risk factor. Both BZA/CE doses induced better changes on the lumbar spine and total hip BMD than placebo after one year, with significant differences being observed at 6 and 12 months (*p* < 0.05). Also, BZA 20 mg/CE 0.45 mg and BZA 20 mg/CE 0.625 mg induced a more significant reduction from baseline of C-telopeptide and procollagen I N-terminal propeptide (*p* < 0.01). These data indicate a protective effect on bone metabolism as well as on BMD.

### 3.3. Effects on Sleep, Quality of Life, Sexual Function, and Treatment Satisfaction

The impact of the TSEC on sleep parameters, quality of life, and treatment satisfaction were assessed in two clinical trials [43, 44], using different tests, including the Medical Outcomes Study (MOS) Sleep Scale. The MOS Sleep Scale is composed of 12 items that assess sleep parameters with six subscales: sleep adequacy, sleep disturbance, sleep quantity, daytime somnolence, snoring, and waking with shortness of breath or a headache. The questionnaire also examines the average time taken to fall asleep (the score is calculated on a 6-point scale: 1 = “all of the time” and 6 = “none of the time”). The domains of the MOS Sleep Scale were examined by comparing treatment responders (subjects with at least 75% improvement from baseline in the average number of moderate-to-severe daily HFs) and nonresponders. The impact on the quality of life (QoL) was assessed using the Menopause-Specific Quality of Life (MSQoL), a questionnaire comprising 29 items with four domains: vasomotor, psychosocial, physical, and sexual function. The MSQoL score is calculated as a mean of several items that range from 1 to 8 (where a maximum score corresponds to the most bothersome symptom). Treatment satisfaction was assessed with the Menopause Symptoms Treatment Satisfaction Questionnaire (MS-TSQ), comprising eight questions related to control of HFs during the day and control of HFs and sweats occurring during the night, sleep, mood, libido, ability to concentrate, medication tolerability, and overall satisfaction. Answers were scored from “extremely satisfied” to “extremely dissatisfied” on a 5-point scale.

Improvements were reported for the time to fall asleep, sleep disturbance, and adequacy for both doses (BZA 20 mg/CE 0.45 mg and BZA 20 mg/CE 0.625 mg) at week 12 in comparison to placebo in the substudy SMART-5 (*p* < 0.05) [43]. In this study, both sleep and QoL were assessed. Also, significant improvements in sleep parameters were seen among responders treated with both doses of BZA/CE at week 12 in comparison with placebo in the trial SMART-2 (*p* < 0.05) [43].

Women being treated either with BZA 20 mg/CE 0.45 mg or BZA 20/CE 0.625 mg showed a significant improvement in QoL parameters at 1-year follow-up in the SMART-5 trial and 12 weeks in the SMART-2 (*p* < 0.001) and SMART-3 (*p* > 0.001) trials [42, 43]. In the study SMART-3 [44], the effect of TSEC on QoL and sexual function was assessed. In this 12-week double-blind, placebo-controlled trial, conducted in 652 postmenopausal women with intact uterus and moderate-to-severe vulvar/vaginal atrophy, patients were randomized to treatment with daily BZA 20 mg/CE 0.45 mg, BZA 20 mg/CE 0.625 mg, BZA 20 mg, or placebo. At week 12, both BZA/CE doses induced a significant improvement in lubrification from baseline according to the Arizona Sexual Experiences (ASEX) Scale compared to placebo (*p* < 0.05). In addition, at 12 weeks, the MENQOL questionnaire showed significant improvements in vasomotor function, sexual function, and total score with BZA 20 mg/CE 0.45 and BZA 20 mg/CE 0.625 mg (*p* < 0.001). MS-TSQ results showed that patients treated with BZA/CE were overall more satisfied with treatment as well as with the day and night control of HFs, quality of sleep, mood, and emotions compared with patients treated with BZA 20 mg or placebo (*p* < 0.05 for all comparisons).

### 3.4. Effects on Vaginal Tissue

Data from vaginal smears showed that the TSEC significantly increased the number of superficial and intermediate cells while decreasing the percentage of parabasal cells, which displays an estrogen-like action that can improve menopausal vaginal atrophy. In the SMART-1 trial [35], a randomized, multicenter, double-blind, placebo-, and active-controlled trial, carried out in 3397 menopausal women with an intact uterus (mean age: 56 years; range: 40–75 years) for 2 years, the groups treated with BZA 20 mg/CE 0.45 and BZA 20 mg/CE 0.625 mg showed a significant increase in the number of superficial and intermediate cells and a reduction in the number of parabasal cells (*p* < 0.001 versus placebo).

The action of the TSEC on vaginal tissue was also studied in the multicenter, double-blind, randomized, placebo-, and active-controlled trial SMART-3 trial [36]. The objective of this study was to compare the efficacy and safety of two doses of BZA/CE versus placebo for the treatment of moderate-severe menopause-associated VVA. The SMART-3 trial enrolled 664 healthy menopausal women (age range: 40–65 years) who were randomized to the following treatment groups: daily BZA 20 mg/CE 0.625 mg, BZA 20 mg/CE 0.45 mg, BZA 20 mg, or placebo during 12 weeks. VVA-related parameters were assessed at screening, week 4, and week 12. Adverse events were recorded during the entire period of study. Groups treated with BZA 20 mg/CE 0.625 mg and BZA 20 mg/CE 0.45 mg showed more superficial cells and fewer parabasal cells in comparison with placebo (*p* < 0.01). Also, significant improvement in vaginal dryness was observed for both groups treated with BZA/CE compared to placebo (*p* < 0.05). Adverse events were not different between groups. The authors concluded that BZA/CE was efficient for the treatment of moderate/severe VVA in menopausal women.

### 3.5. Effects on Endometrial Tissue

The action of the TSEC on the endometrium was evaluated in the randomized, double-blind, placebo-, and active-controlled (raloxifene 60 mg) SMART-1 trial [45]. The drugs investigated in SMART-1 were BZA (10, 20, or 40 mg) in combination with CE (0.625 or 0.45 mg), daily raloxifene, or placebo for two years. The study population was composed of healthy postmenopausal women (*n* = 3397), aged from 40 to 75 years, with an intact uterus and a body mass index ≤ 32.2 kg/m^2^. The primary endpoint was the incidence of endometrial hyperplasia at 12 months. Endometrial biopsies were performed at screening and months 6, 12, and 24; biopsies were evaluated by two pathologists blinded to treatment; transvaginal ultrasounds were carried out to assess endometrial thickness, ovarian volume, and the presence of ovarian cysts, at the screening and months 12 and 24. Treatment with BZA 20 mg/CE 0.625 mg or BZA 20 mg/CE 0.45 mg was associated with a low rate (<1%) of endometrial hyperplasia that was not significantly different from that observed with placebo during the 24 months of the study. Also, the endometrial thickness in patients treated with BZA (20 or 40 mg)/CE (0.625 or 0.45 mg) was not significantly different from patients treated with placebo. Groups treated with BZA 10 mg/0.625 mg CE or BZA 10 mg/CE 0.45 mg showed an increased number of hyperplasia cases. Thus, the lowest effective dose of BZA capable of significantly inhibit the endometrial-stimulating effect induced by CE 0.45 mg and 0.625 was 20 mg/day. This study showed that, while administering CE to patients, endometrial protection can be achieved through the modulation of ER by BZA. In the SMART-5 trial [41], the endometrial effects of BZA/CE were also studied: hyperplasia rate was <1% and no significant differences were observed in comparison with placebo after 12 months of BZA 20 mg/CE 0.45 mg treatment.

### 3.6. Effects on Uterine Bleeding

The observed endometrial action of the TSEC indicates an optimal balance between its two components, BZA 20 mg/CE 0.45 mg or BZA 20 mg/CE 0.625 mg. This balance is also displayed by the high percentage of amenorrhea observed in patients. Indeed, in the SMART-1 and SMART-5 trials [42, 46], the cumulative amenorrhea rate during the first year of treatment was 83% for patients treated with BZA 20 mg/CE 0.45 mg, a rate similar to that observed for placebo-treated patients (SMART-1). Furthermore, treatment with BZA 20 mg/CE 0.45 mg induced a high percentage of amenorrhea that was different from that obtained with CE 0.45 mg/medroxyprogesterone acetate 1.5 mg (*p* < 0.001) in 13 observation cycles [42]. Bleeding-related adverse events were significantly lower in BZA 20 mg/CE 0.45 mg and BZA 20 mg/CE 0.625 mg groups (7% and 5.7%, resp.) compared to those reported for treatment with CE 0.45 mg/MPA 1.5 mg (22.3%) (overall *p* < 0.001) [42].

### 3.7. Effects on Breast Tissue

The action of the TSEC on breast tissue was evaluated in the randomized, double-blind, placebo-, and active-controlled (CE/MPA) SMART-5 trial [39]. The SMART-5 investigated the effect of BZA/CE on mammographic density and other breast parameters in a substudy that enrolled 940 women with technically acceptable digital mammograms at screening and 12 months. The treatments under study were BZA 20 mg and CE 0.45 or 0.625 mg, placebo, BZA 20 mg, and CE (0.45 mg) + MPA (1.5 mg). Mammograms were read centrally by a single radiologist blinded to treatment and time sequence of exams; percent breast density was determined using a validated software. The noninferiority of treatments was based on a predefined margin of 1.5% for a comparison of adjusted breast density mean differences at 12 months. BZA 20 mg and CE 0.45 mg or 0.625 mg demonstrated noninferiority compared to placebo in respect to breast density. Mammographic density decreased from baseline with BZA 20 mg/CE 0.45 (−0.38%), BZA 20 mg/CE 0.625 mg (−0.44%), and placebo (−0.32%) and significantly increased from baseline with CE 0.45 mg/MPA 1.5 mg (+1.60%, *p* < 0.001 versus placebo). Both doses of BZA/CE showed breast tenderness rates (expressed as the percentage of women with breast tenderness for one or more days) similar to placebo and significantly lower than those induced by CE 0.45 mg/MPA (*p* < 0.001). Furthermore, no differences in adverse events between groups were reported. Breast cancer rates were low and with no significant differences among groups: BZA 20 mg/CE 0.45 mg, 0.4%; BZA 20/CE 0.625, 0%; MPA 1.5/CE 0.45, 0.5%; placebo, 0.2%. In conclusion, in this study, BZA 20 mg and CE 0.45 mg or 0.625 mg did not increase mammographic density nor breast tenderness during one year of follow-up and presented a favorable safety profile for breast tissue.

In another trial [47], a randomized, placebo-, and active-controlled trial carried out on mammograms from 507 women (age range: 55.2–56.3 years) evaluated the effect of BZA 20 mg/CE 0.45 mg, BZA 20 mg/CE 0.625 mg, raloxifene 60 mg, and placebo on breast tissue for two years. Mammograms were also read by a radiologist blinded to treatment and to the date in which mammograms were performed; changes in breast density were also assessed with validated software. Mean mammographic density changes from baseline at 24 months were the following: BZA 20 mg/CE 0.45 mg, −0.39%; BZA 20 mg/CE 0.625 mg, −0.05%; raloxifene 60 mg, −0.23%; placebo, −0.42%. Reductions in mammographic density from baseline were statistically significant for BZA 20 mg CE 0.45 mg and placebo. Based on these data, treatment with BZA/CE up to 2 years did not increase mammographic density or breast tenderness, and there was no evidence of an increased breast cancer risk, which suggests that TSEC has a better breast-related safety profile than traditional hormone replacement therapies [48]. These outcomes may be related to the fact that TSEC is the first hormonal treatment for menopause without progestin. Neither conjugated estrogens alone (contrary to the formulation conjugated estrogens plus progestin) nor BZA alone demonstrated breast cancer increased risk in previous studies [49–51].

### 3.8. Effects on Lipid and Coagulation Parameters

In the SMART-1 trial [35], carried out in healthy postmenopausal women with an intact uterus (*n* = 3397, aged from 40 to 75 years), LDL cholesterol levels decreased, and HDL cholesterol levels increased during two years of treatment with the TSEC. Overall, the impact on coagulation parameters (antithrombin III, protein C, protein S, fibrinogen, partial thromboplastin time, prothrombin time, and D-dimer) and lipid parameters did not show clinically significant changes. A pooled analysis of data from the SMART trials confirmed these results [48]. After analyzing data obtained with BZA 20 mg/CE 0.45 mg, BZA 20 mg/CE 0.625 mg, and placebo for 12 months, the authors observed that BZA 20 mg/CE 0.45 mg and BZA 20 mg/CE 0.625 induced significant improvements in total cholesterol (−4.20 and −4.37% versus −0.88%), LDL cholesterol (−9.33 and −10.78% versus −1.08%), HDL cholesterol (4.59 and 6.21% versus 1.30%), and LDL/HDL ratio LDL/HDL (−11.59 and −14.00% versus −0.84%) compared to placebo. On the other hand, triglycerides were increased from baseline with both doses (*p* < 0.001) in comparison with placebo (15.13% and 15.74% versus 4.43%) as generally reported with the oral administration of estrogen, but this aspect is considered not clinically relevant. These trends were confirmed by the results obtained at 24 months. Thus, the TSEC is associated with predominantly favorable changes in lipid parameters for up to 2 years of treatment in postmenopausal women.

### 3.9. Cardiovascular Effects

Cardiovascular effects were assessed in the SMART-1 trial performed on healthy postmenopausal (for at least 1 year) women (*n* = 3397) age 40–75 with an intact uterus (body mass index ≤ 32.2 kg/m^2^) [35]. The incidence of cardiovascular adverse events was low (<1%) across treatment groups and with no significant differences. BZA 20 mg/CE 0.45 mg and BZA 20 mg/CE 0.625 mg did not show differences in the incidence of myocardial infarction, coronary artery disease, coronary artery insufficiency, and venous thromboembolic events compared to placebo after a 2-year follow-up [35]. The incidence of these conditions after two years was as follows:

- Risk of acute myocardial infarction (AMI) with BZA/CE versus PBO = 0.48 (CI 95%: 0.05–4.66)
- Risk of coronary artery disease (CAD)/coronary artery insufficiency with BZA/CE versus PBO = 1.29 (CI 95%: 0.16–10.34)
- Risk of venous thromboembolism (VTE) with BZA/CE versus PBO (RR = 0.48; CI 95%: 0.05–4.66)

It is noteworthy that not only there is no increase in artery disease but also venous disease is not increased after two years of treatment with the TSEC. In addition, body weight increased by less than 0.9 kg and BMI by less than 0.4 kg/m2 with BZA 20 mg/CE 0.45, BZA 20 mg/CE 0.625, and placebo at 3, 6, 12, and 24 months of follow-up [52], which suggests a neutral metabolic impact.

A meta-analysis of all five SMART trials [53] evaluated cardiovascular safety data from healthy postmenopausal women that were given at least one dose of BZA 20 mg/CE 0.45 mg (*n* = 1585) and BZA 20 mg/CE 0.625 mg (*n* = 1583), any dose of BZA/CE (*n* = 4868) or placebo (*n* = 1241) for a follow-up period of up to 2 years. Events of venous thromboembolism (VTE), coronary artery disease (CHD), and cerebrovascular events (stroke) were assessed. Rates per 100 women/year are presented in Table 4.

For any dose of BZA/CE, the relative risk (CI 95%) of VTE was 0.5 (0.1–1.8), of stroke was 0.5 (0.1–2.6), and of coronary disease was 0.63 (0.23–1.74), with no significant differences with placebo. This meta-analysis indicates that for up to 2 years of treatment, BZA 20 mg/CE 0.45 mg and BZA 20 mg/CE 0.625 mg present an acceptable cardiovascular safety profile, with rates of stroke and coronary artery disease similar to placebo in healthy postmenopausal women. Also, the risk of VTE was low [53].

## 4. Conclusion: Role of the TSEC in the Management of Menopausal Patients

Overall, data from the literature demonstrates that the TSEC

- significantly reduces vasomotor symptoms and increases the quality of sleep (improving the quality of life),
- protects bone tissue,
- improves vaginal atrophy,
- does not stimulate breast tissue,
- does not stimulate endometrial tissue,
- does not increase cardiovascular risk.

Considering the clinical pharmacology characteristics of a TSEC, this novel drug class, particularly the combination BZA 20 mg/CE 0.45 mg, may represent the first choice in the treatment of patients with severe symptoms during the early phases of menopause. This conclusion is also based on breast- and uterus-related safety data obtained so far and on the positive effects on vaginal atrophy and bone tissue that should be considered when providing menopausal women with a personalized therapy.

## Figures and Tables

**Table 1:** Targeted responses to systemic menopause treatments [6–11].

Target	SERMs Ral, BZA	Estrogen	STEAR (tibolone)	TSEC (BZA/CE)
Uterus	0	−	=	0
Hot flushes	−	+	+	+
Vagina	−	+	+	+
Bone	+	+	+	+
Breast	+	0	=	0
Sexual function	=	+	++	+
Energy levels	=	+	++	+

STERM: selective estrogen receptor modulator; Ral: raloxifene; BZA: bazedoxifene; STEAR: selective tissue estrogenic activity regulator (tibolone); TSEC: tissue selective estrogen complex = BZA + CE; +: positive effect; ++: very positive effect; −: negative effect; =: neutral effect.

**Table 2:** Preclinical studies on breast tissue.

	Model	Results
Song et al. [23]	MCF-7 breast cancer cells	(i) BZA blocked CE-induced stimulation, including DNA synthesis, reduction of apoptosis, expression of cMyc, pS2, and WNT1-inducible signaling pathway protein 2
Wardell et al. [24]	MCF-7 breast cancer cells	(i) BZA showed inverse agonist activity on many genes regulated by estradiol
Chang et al. [25]	MCF-7 breast cancer cells and microarrays	(i) BZA, RLZ, and LAS inhibited the proliferation of breast cancer cells induced by CE, with the following antagonist power: BZA > RLX > LAS (ii) BZA inhibited a group of genes regulated by CE; this profile is different from those of RLX and LAS
Peano et al. [26]	Ovariectomized mice	(i) The stimulating effects of CE on the expression of amphiregulin (a marker of ductal proliferation) were antagonized by BZA > RLX > LAS (ii) BZA was more effective than RLX and LAS in reducing ductal growth
Song et al. [23]	Ovariectomized mice	(i) BZA blocked gene expression induced by CE and the growth of mammary terminal ducts and acini (ii) BZA blocked tumor growth and gene expression in mice with MCF-7 xenografts
Ethun et al. [27]	Ovariectomized cynomolgus macaques	(i) 6-month treatment with BZA/CE significantly reduced the increase in epithelial density, the growth, and the ductal proliferation induced by CE (all *p* < 0.05) (ii) BZA/CE treatment reduced ER protein expression and activity markers

**Table 3:** Preclinical studies on endometrial tissue.

	Model	Results
Peano et al. [26]	Ovariectomized mice	(i) BZA, in contrast to RLX or LAS, antagonized the increase in uterine wet weight to levels similar to that induced by vehicle
Oliva et al. [28]	Ovariectomized mitosis—luciferase mice	(i) BZA and CE completely inhibited the proliferative effects of CE in the uterus
Kharode et al. [29]	Ovariectomized rats	(i) BZA/CE inhibited CE-induced uterine stimulation
Komm and Lyttle [22]	Ovariectomized rats	(i) BZA/CE reduced CE-induced uterine wet weight increase in a dose-dependent manner
Ethun et al. [30]	Ovariectomized cynomolgus macaques	(i) Endometrial and epithelial proliferations were significantly reduced with BZA/CE in comparison with CE alone (*p* < 0.001) (ii) Endometrial hyperplasia rate following BZA/CE treatment was comparable to controls

**Table 4:** Cardiovascular events (venous thromboembolism, stroke, and coronary artery disease) reported in SMART trials (data presented as incidence per 1000 women/year, CI 95%) [53].

Dose	Venous thromboembolism	Stroke	Coronary artery disease
BZA 20 mg/CE 0.45 mg	0.3 (0.0–2.0)	0.4 (0.0–2.4)	2.6 (0.0–5.6)
BZA 20 mg/CE 0.625 mg	0 (0.0–1.5)	0.2 (0.0–1.9)	1.4 (0.0–3.9)
BZA/CE all doses	0.7 (0.0–1.5)	0.44 (0.0–1.1)	2.4 (1.0–3.7)
Placebo	0.6 (0.0–2.9)	0.0 (0.0–1.7)	2.0 (0.0–5.2)
